# Supplementary material for: Crystal structure of the Na+/H+ antiporter NhaA at active pH reveals the mechanistic basis for pH sensing
Source: Nat Commun. 2022 Oct 26;13:6383. doi: 10.1038/s41467-022-34120-z (PMC9606361; doi:10.1038/s41467-022-34120-z)

Supplementary Table 1. NhaA mutations in the cytoplasmic pH sensor shown to shift the wildtype pH for activation.

Mutation	pH shift	Magnitude (pH)	Reference (doi)
Asn64Cys	Acidic	not assesed	10.1074/jbc.M109.047134
Asp65Cys	Basic	0.5	10.1074/jbc.M109.047134
Leu67Cys	Basic	1	10.1074/jbc.M109.047134
Glu78Cys	Basic	1	10.1074/jbc.M109.047134
Glu82Cys	Basic	1	10.1074/jbc.M109.047134
Glu241Cys	Acidic	0.3	10.1074/jbc.274.35.24617
Lys242Cys	Basic	0.5	10.1074/jbc.M800482200
Arg250Cys	Basic	0.5	10.1074/jbc.M800482200
Glu252Cys	Basic	1	10.1074/jbc.M309021200
His253Cys	Basic	1	10.1074/jbc.M800482200
His256Cys	Basic	0.5	10.1074/jbc.M800482200
Val254Cys	Acidic	1	10.1074/jbc.274.35.24617

Supplementary Table 2. Protonation states in MD simulations. **MD protonation state** is the label for the specific fixed set of protonation state of the titratable residues in NhaA. All residues (except the explicitly listed ones D163, D164, and K300) were protonated according to the **simulation effective pH** and the pK_a (from Huang et al. [1]). The **funnel total charge** is the total charge of residues D11, E78, R81, E82, D133, K153, H243, K249, R250, E252, H253, and H256. The **binding site effective pH** is the pH at which the binding site residues **D163, D164**, and **K300** would be predominantly found in the given protonation state combination[1]. The **binding site total charge** is the sum of the charges of the binding site residues. "–" indicates the deprotonated form of the binding site residue while "H+" indicates a simulation with the protonated form.

MD protonation state	simulation effective pH	funnel total charge	binding site effective pH	binding site total charge	D163	D164	K300
S1	7.5	−1	4	0	−	H+	H+
S2	7.5	−1	8	−1	−	−	H+
S4	7.5	−1	11.5	−2	−	−	−
S3	7.5	−1	2.5	+1	H+	H+	H+
low pH	3.5	+3	4	0	−	H+	H+

Supplementary Table 3. MD simulations. **state** is the protonation state configuration of D163, D164, K300 as described in the text and Table S3; **run** numbers independent repeat simulations with total run time, number of atoms, and initial orthorhombic system size. The **binding probability** in protomer A and B was computed as the fraction of total simulation time during which a Na^+ was bound to either D164 or D163. **continued from** indicates if the simulation was run continuously from the equilibration period or interrupted and restarted after bound Na^+ ion was exchanged against a random bulk water molecule.

ID	state	run	GROMACS version	runtime (ns)	natoms	box size (nm)	binding probability A	binding probability B	continued from	Na+ site
S1_0	S1	0	2018.2	1000	134662	12.1 12.1 9.0	0	0	equilibration	
S1_1	S1	1	2018.2	1000	134662	12.1 12.1 9.0	0	0	equilibration	
S1_2	S1	2	2018.2	1000	134662	12.1 12.1 9.0	0	0	equilibration	
S2_0-pre	S2	0	2018.1	649	146241	11.9 11.9 10.0	0.998	0.996	equilibration	
S2_0-post	S2	0	2018.1	654.1	146241	11.9 11.9 10.0	0.987	0.998	S2_0-pre	bound ion/water swap
S2_1-pre	S2	1	2018.1	638.2	146241	11.9 11.9 10.0	0.981	0.998	equilibration	
S2_1-post	S2	1	2018.1	914.1	146241	11.9 11.9 10.0	0.956	0.976	S2_1-pre	bound ion/water swap
S2_2-pre	S2	2	2018.1	579.5	146241	11.9 11.9 10.0	0.992	0.998	equilibration	
S2_2-post	S2	2	2018.1	575.8	146241	11.9 11.9 10.0	0.992	0.99	S2_2-pre	bound ion/water swap
S3_0	S3	0	2018.1	1000	146241	12.0 12.0 10.0	0	0	equilibration	
S3_1	S3	1	2018.1	1000	146241	12.0 12.0 10.0	0	0	equilibration	
S3_2	S3	2	2018.1	1000	146241	12.0 12.0 10.0	0	0	equilibration	
S4_0	S4	0	2018.1	1000	146237	12.0 12.0 10.0	0.995	0.345	equilibration	
S4_1	S4	1	2018.1	1000	146237	12.0 12.0 10.0	0.999	0.997	equilibration	
S4_2	S4	2	2018.1	1000	146237	12.0 12.0 10.0	0.999	0.997	equilibration	
lowpH_1	low pH	1	2018.8	793.7	130998	12.3 12.3 8.4	0	0	equilibration	
lowpH_2	low pH	2	2018.8	820.5	130998	12.3 12.3 8.4	0	0	equilibration	
lowpH_3	low pH	3	2018.8	837.8	130998	12.3 12.3 8.4	0	0	equilibration	

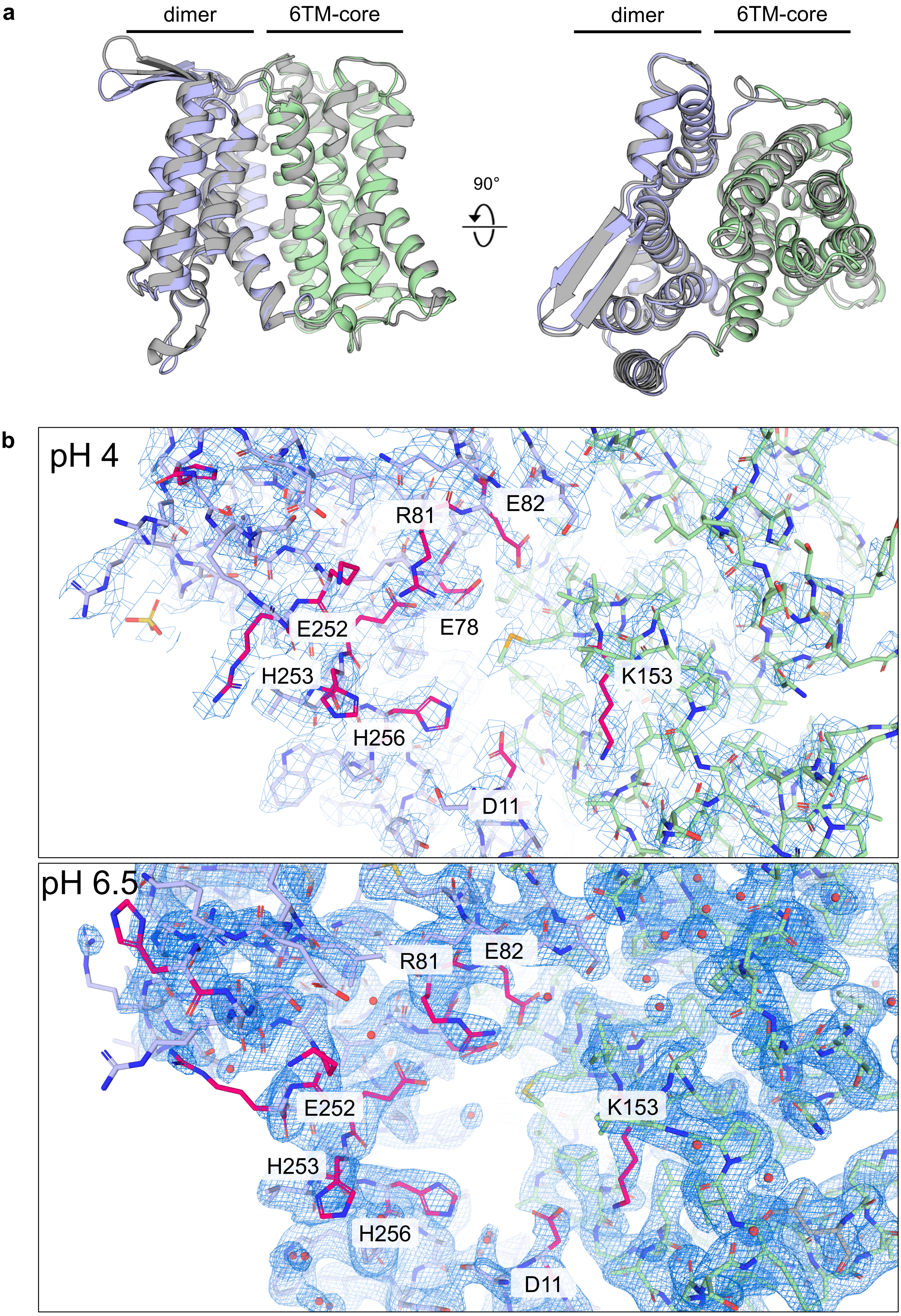
a
dimer
6TM-core
90°
dimer
6TM-core
b
pH 4
R81
E82
E252
E78
H253
K153
H256
D11
pH 6.5
R81
E82
E252
K153
H253
H256
D11

Supplementary Figure 1. Structural comparison of *Ec*NhaA crystal structures. a. Overlay of cartoon representation of the *Ec*NhaA lipidic cubic phase (LCP) structure at 2.2 Å (monoLCP-NhaA) (coloured, PDB ID: 7S24 [https://www.rcsb.org/structure/7S24]) and the previously solved 3.5 Å *Ec*NhaA vapour diffusion structure (monoVD-NhaA) (grey, PDB ID: 1ZCD [https://www.rcsb.org/structure/1ZCD]). **b.** Top panel: Electron density map 2Fo − Fc (1.5σ) (blue mesh) on the NhaA triple mutant dimer structure at pH 4.0 (PDB ID: 4ATV [https://www.rcsb.org/structure/4atv] with pH gating residues shown as pink sticks and labelled. Bottom panel: Electron density map 2Fo − Fc (1.5σ) (blue mesh) on the NhaA triple mutant monomeric structure determined here at pH 6.5 (PDB ID: 4ATV [https://www.rcsb.org/structure/4atv]) with pH gating residues shown as pink sticks and labelled (PDB ID: 7S24 [https://www.rcsb.org/structure/7S24]).

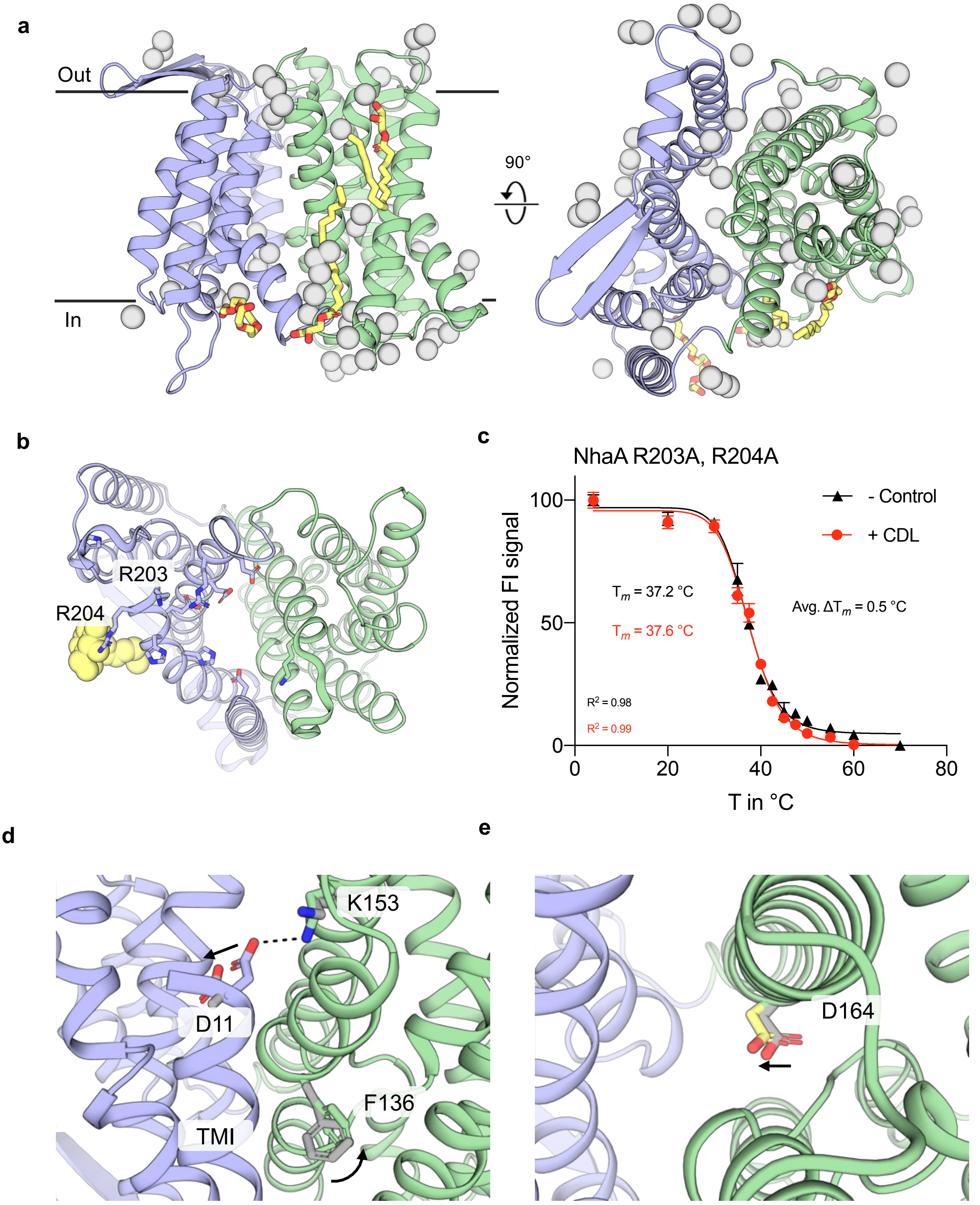

Supplementary Figure 2. Lipids interactions in NhaA and pH induced changes. a. Cartoon representation of the monomeric *Ec*NhaA LCP crystal structure (monoLCP-NhaA). The dimer domain is depicted in blue, the core transport domain in green, crystal waters in grey spheres, and lipids in yellow sticks. Side-view (left) and top-view (right). **b.** Cartoon representation of the here presented 2.2 Å NhaA structure from the periplasmic site. The detergent molecule (yellow) from the NhaA dimer (PDB ID: 4ATV [https://www.rcsb.org/structure/4atv] is shown in sphere representation, located at the proposed cardiolipin binding site. **c.** Thermostability of proposed *Ec*NhaA lipid-binding residue double-mutant Arg203Ala, Arg204Ala without (black)

and in presence (red) of cardiolipin (CDL). Data presented are normalized fluorescence as mean values ± SEM of n = 3 technical repeats; the apparent T_m was calculated with a sigmoidal 4-parameter logistic regression function. Average ΔT_m from n = 2 independent experiments. **d.** Cartoon representation of NhaA, showing the coupling between the Asp11-Lys153 interaction and Phe136 between the high pH (PDB ID: 7S24 [https://www.rcsb.org/structure/7S24], coloured) and low pH structure (PDB ID: 4AU5 [https://www.rcsb.org/structure/4AU5], grey). **e.** Cartoon representation of NhaA, showing the movement of Asp164 between the high pH (PDB ID: 7S24 [https://www.rcsb.org/structure/7S24], coloured) and low pH structure (PDB ID: 4AU5 [https://www.rcsb.org/structure/4AU5], grey). Source data are provided as a Source Data file.

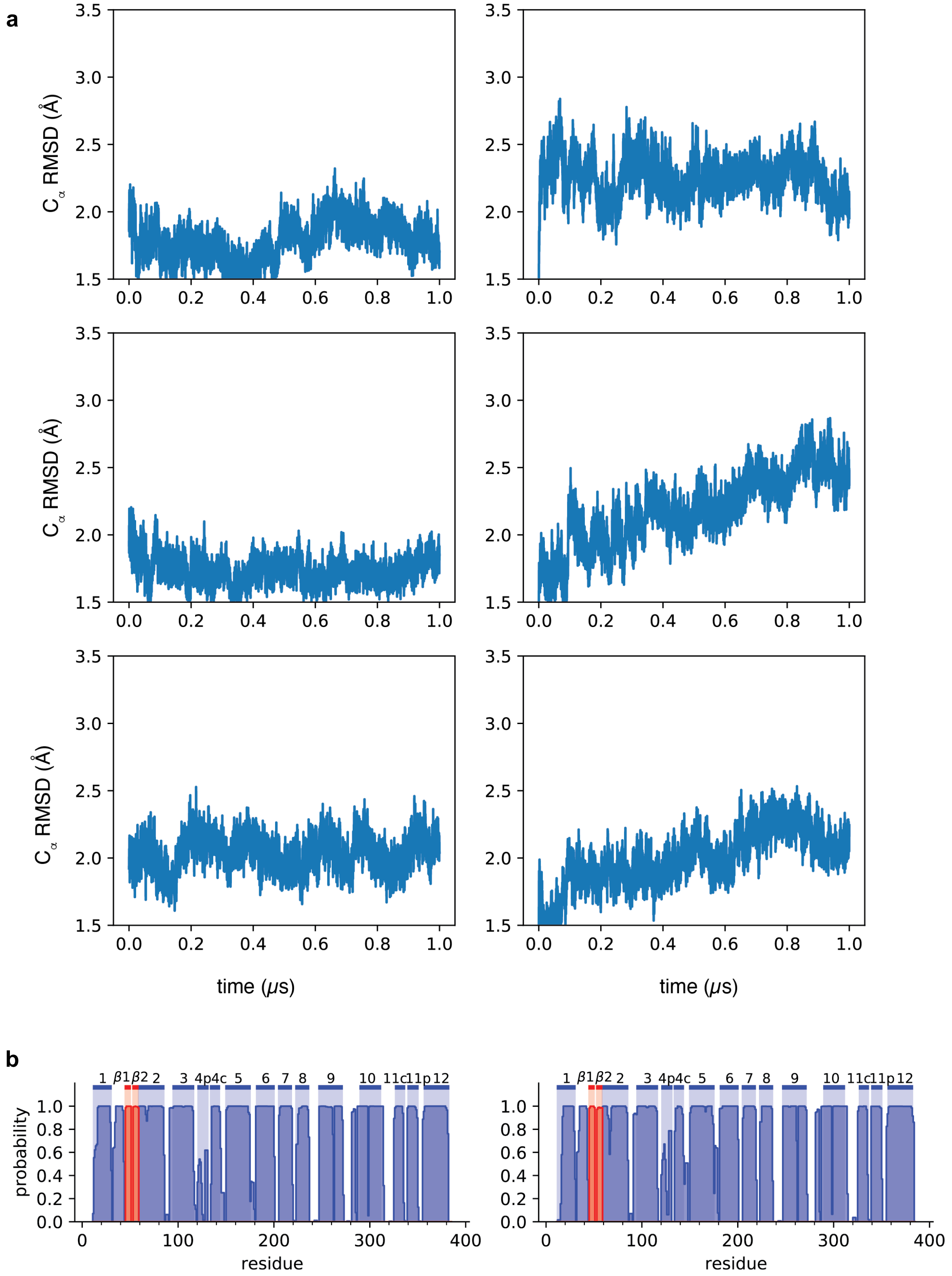

Supplementary Figure 3. Structural stability of the active state crystal structure when simulated under high pH (pH 7.5) conditions with S4 protonation states. a. C-alpha RMSD for each protomer (left, protomer A; right, protomer B) with the crystal structure as reference. Each row is an independent repeat simulation. (*top*: S4_0, *middle*: S4_1, *bottom*: S4_2, see Supplementary Table S4 for details on the simulations) **b.** Probability to observe a specific secondary structure (blue: alpha helix, red: beta sheet) for any residue, averaged over all three repeat simulations for each protomer separately. The secondary structure elements of the crystal

structure are indicated with their numbers (for helices) or names (beta 1/2 for the beta sheets) as bars above the plot.

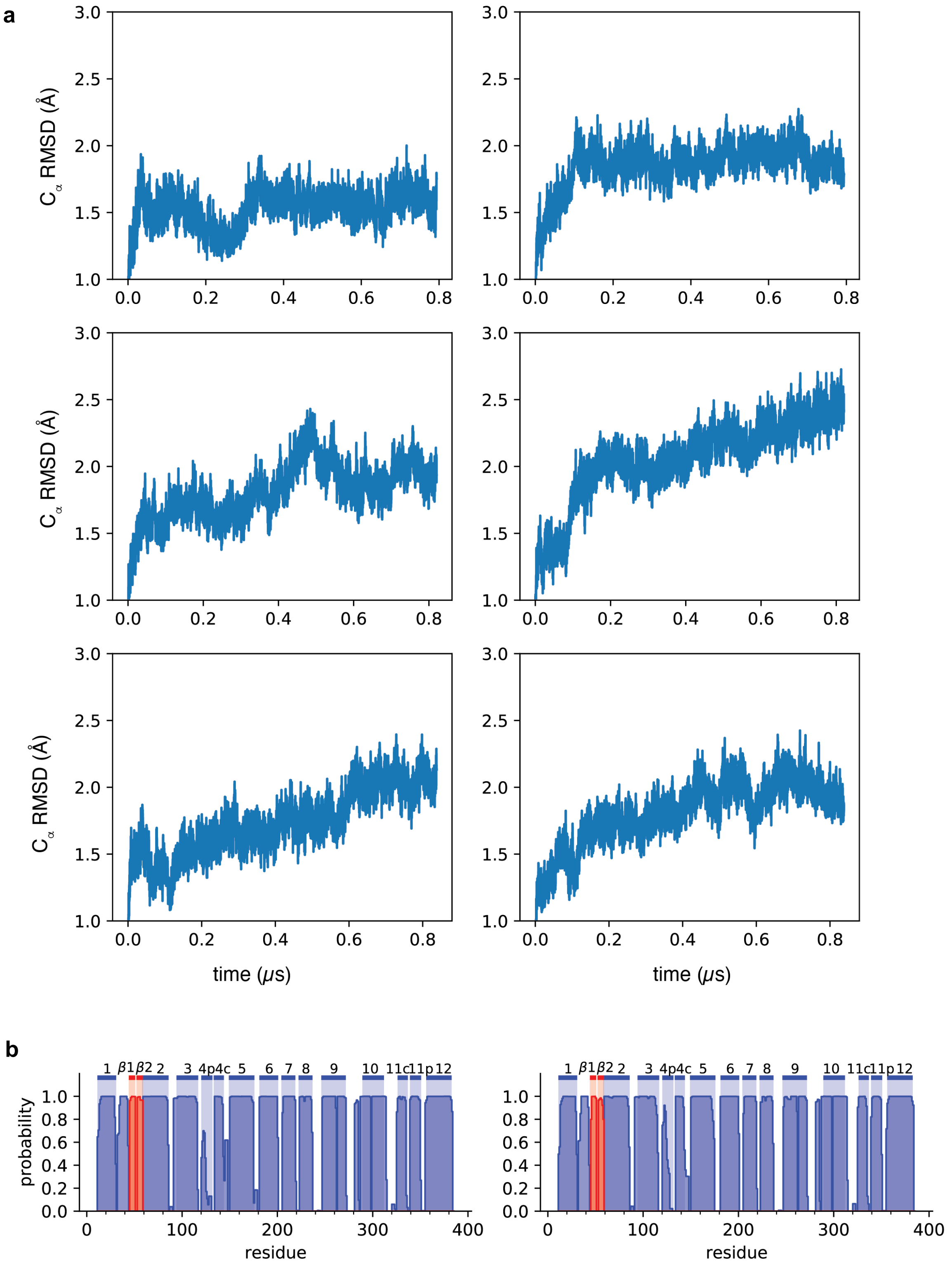

Supplementary Figure 4. Structural stability of the active state crystal structure when simulated under low pH (pH 3.5) conditions. a. C-alpha RMSD for each protomer (left, protomer A; right, protomer B) with the crystal structure as reference. Each row is an independent repeat simulation. (*top*: lowpH_1, *middle*: lowpH_2, *bottom*: lowpH_3, see Supplementary Table S4 for details on the simulations) **b.** Probability to observe a specific secondary structure (blue: alpha helix, red: beta sheet) for any residue, averaged over all three repeat simulations for each protomer separately. The secondary structure elements of the crystal

structure are indicated with their numbers (for helices) or names (beta 1/2 for the beta sheets) as bars above the plot.

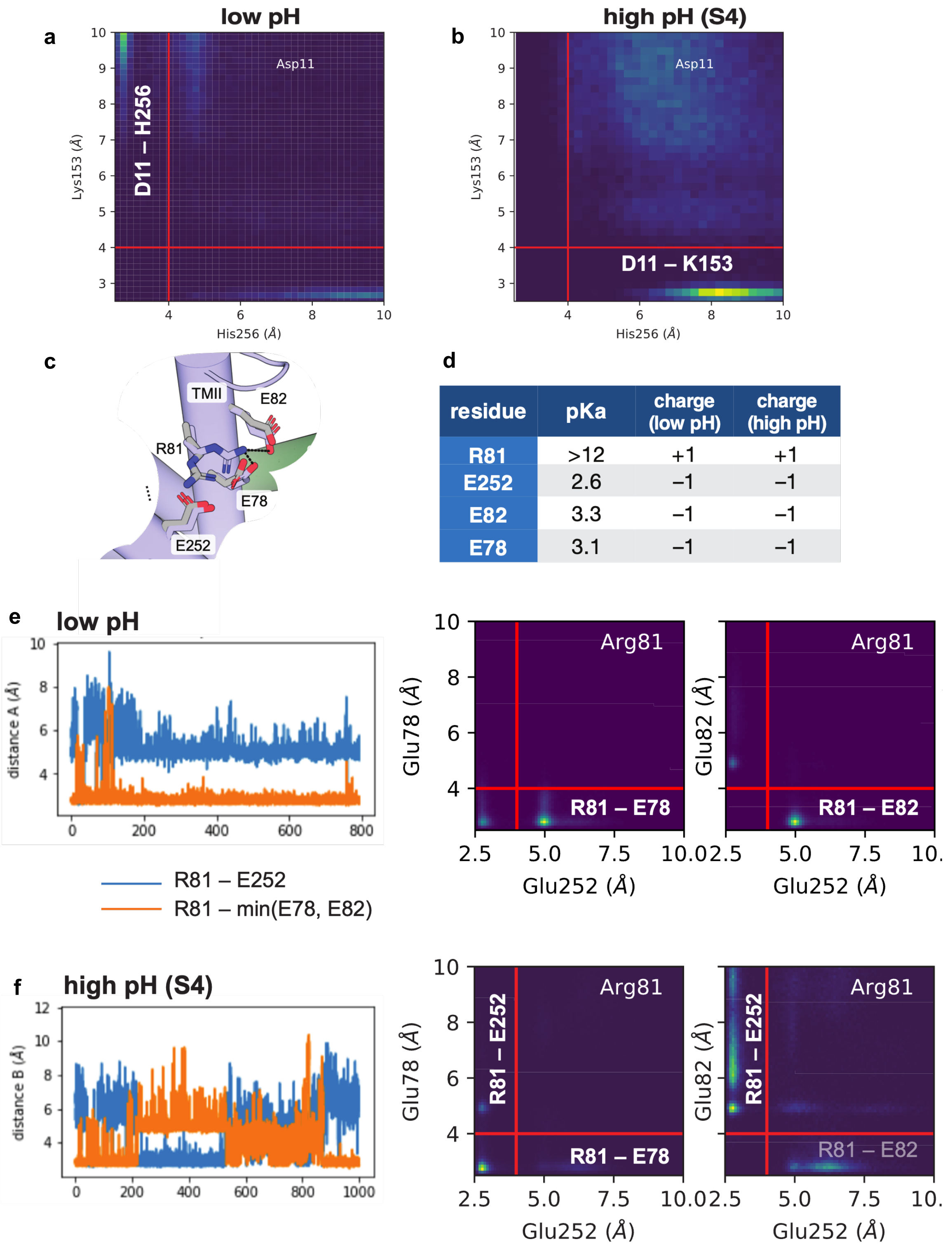

residue	pKa	charge (low pH)	charge (high pH)
R81	>12	+1	+1
E252	2.6	–1	–1
E82	3.3	–1	–1
E78	3.1	–1	–1

Supplementary Figure 5. MD simulations of pH funnel interactions at low and high pH. a, b. Salt bridge switch between Asp11 (D11) and His256 (H256) at low pH (**a**) and between Asp11 and Lys153 (K153) at high pH from the S4 simulations (**b**), shown as 2D probability density distributions of the distance of D11 to H256 and K153. The arbitrary density scale ranges from dark blue (0) to bright yellow (highest probability). Distributions below or left of red lines at 4 Å indicate salt-bridge/H-bond contact, as indicated. Data from all repeat

simulations with the corresponding protonation state were combined. In low pH (**a**), the dominant close contact is H256 < 3 Å, with distance to K156 ≥ 8 Å (although some K156 contacts remain visible, mostly from the initial part of the trajectories, which started from high pH structure). In the high pH (S4) simulations (**b**), the diffuse density above 4 Å indicates states without either close contacts, but high density shows persistent K153-D11 contacts while distance to H256 is > 6 Å (peak at 8 Å). **c.** Salt bridge switch between R81 and E252 and R81 and E78/E82, shown as in Figure 3. **d.** Predicted pKa and charge state at low pH (3.5) and high pH (7.5). The protonation states are not predicted to change. **e.** left panel: Typical timeseries of the distance between R81 and E252 (data from simulation low pH, repeat 1, protomer A) or the shorter of the two distances between R81 and E78 or E82 (data from simulation S4, repeat 1, protomer B). right panel: At low pH, R81 interacts frequently with E78/E82 although salt bridges with E252 are also observed less frequently. **f.** left panel: At high pH, R81 switches interactions between E78/E82 and E252: right panel: at high pH, R81 frequently forms a salt bridge with E78 but can also switch to E252 (simultaneously with E78) or (less frequently) to E82.

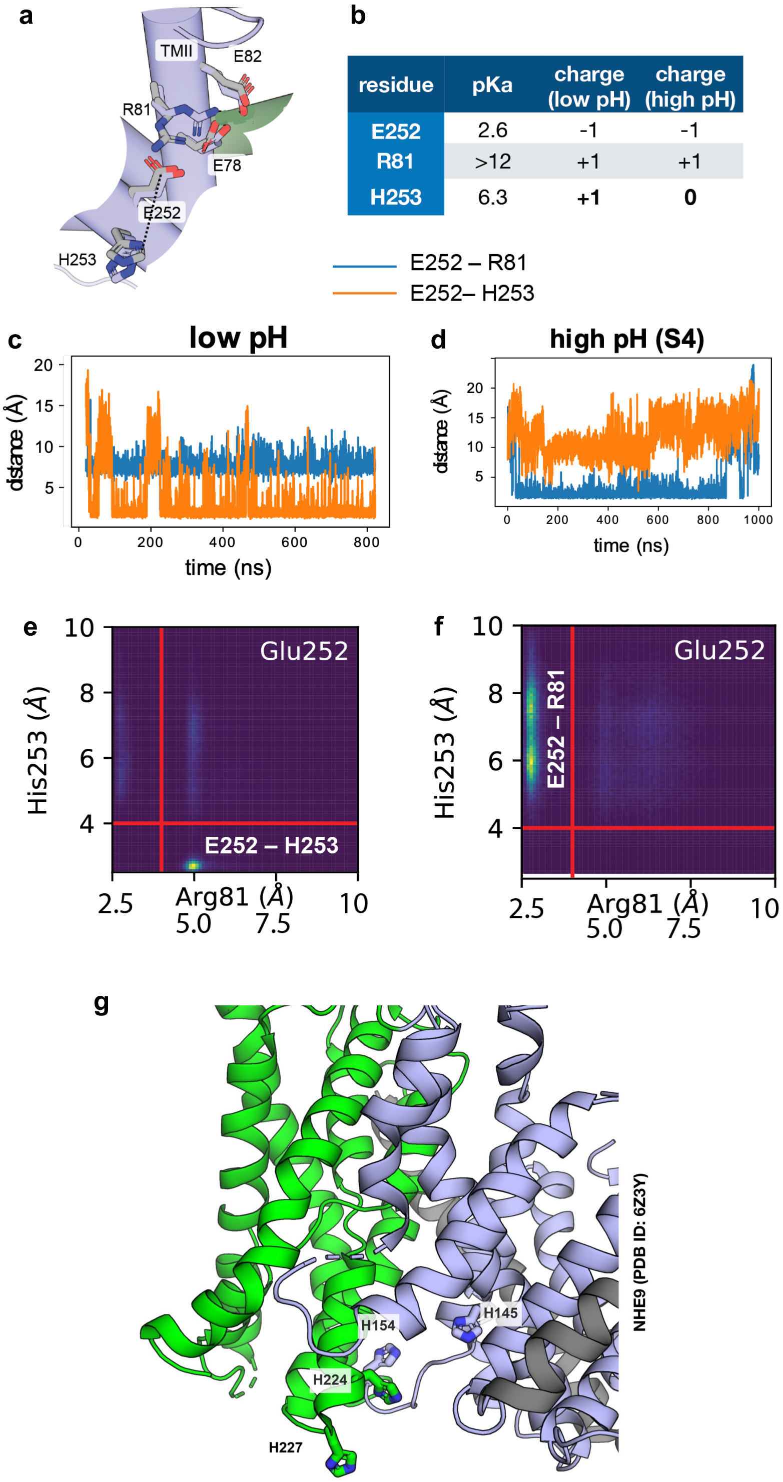

residue	pKa	charge (low pH)	charge (high pH)
E252	2.6	-1	-1
R81	>12	+1	+1
H253	6.3	+1	0

Supplementary Figure 6. MD simulations of pH funnel interactions at low and high pH. a. Salt bridge switch between E252 and R81 and between E252 and H253, shown as in Figure 3. **b.** Predicted pKa and charge state at low pH (3.5) and high pH (7.5). The protonation state of H253 changes between low and high pH. **c, d.** Typical timeseries of the distance between E252 and R81 (data from MD simulation low pH, repeat 2, protomer A) or E252 and H253 (data from simulation S4, repeat 1, protomer A). **e, f.** 2D probability density distribution of the

distance of E252 to R81 and H253. The arbitrary density scale ranges from dark blue (0) to bright yellow (highest probability). Distributions below or left of red lines at 4 Å indicate salt-bridge/H-bond contact. Data from all repeat simulations with the corresponding protonation state were combined. **e.** At low pH, the dominant close contact is E252-H253 although R81 remains not far a distance of 5 Å. **f.** At high pH, E252 and R81 form a salt bridge whereas the E252-H253 distance fluctuates between 5 Å and 10 Å. **g.** Cartoon representation of horse NHE9 inward-facing structure with only one monomer shown, with the core domain (green) and dimerization domain (blue). Labelled histidine residues located around the cavity entrance. His154, His154 and His224 are strictly conserved in NHE9 and either NHE7 or NHE6. The residue His227 is conserved across NHE1, NHE2, NHE3, NHE5, NHE7; NHE8 and NHE9[2].

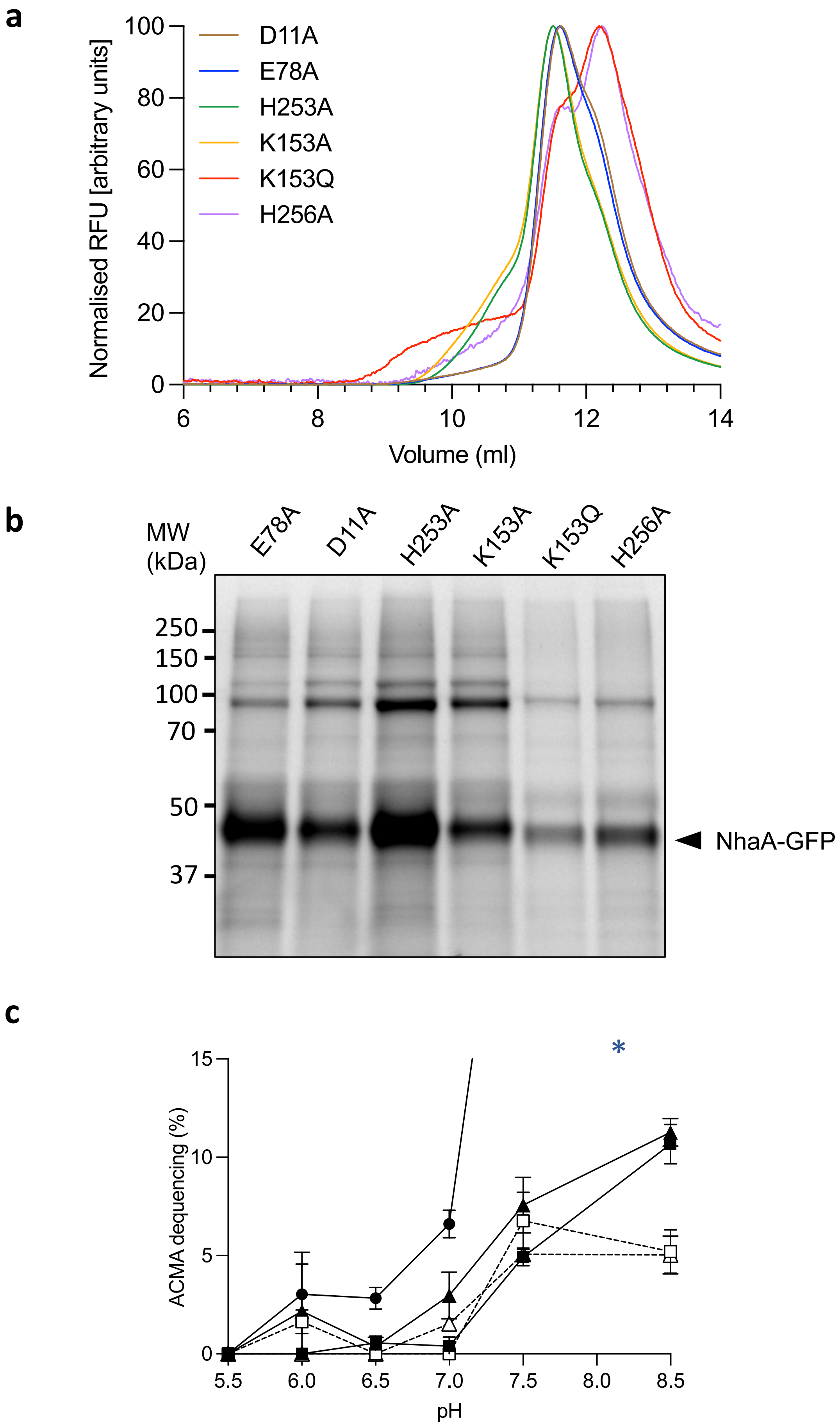

a
Normalised RFU [arbitrary units]
Volume (ml)
D11A
E78A
H253A
K153A
K153Q
H256A
b
MW (kDa)
E78A
D11A
H253A
K153A
K153Q
H256A
250
150
100
70
50
37
NhaA-GFP
c
ACMA dequencing (%)
pH
*

Supplementary Figure 7. Biochemical characterization of NhaA pH gating mutants. a. Fluorescence-detection size-exclusion chromatography (FSEC) traces of purified NhaA variants showing a major monodisperse peak at the same position as NhaA wildtype (see ref [3]). **b.** Purified NhaA-GFP fusions were analysed by SDS-page and Coomassie-staining to show that all variants used in proteoliposome assays had a similar level of purity. **c.** Zoomed in view of the pH dependent profiles shown in Figure 3f. to better show Li^+-H^+ exchange for variants with low activity (ACMA dequenching). Error bars represent SEM from n = 3 technical repeats. 'Source data are provided as a Source Data file.

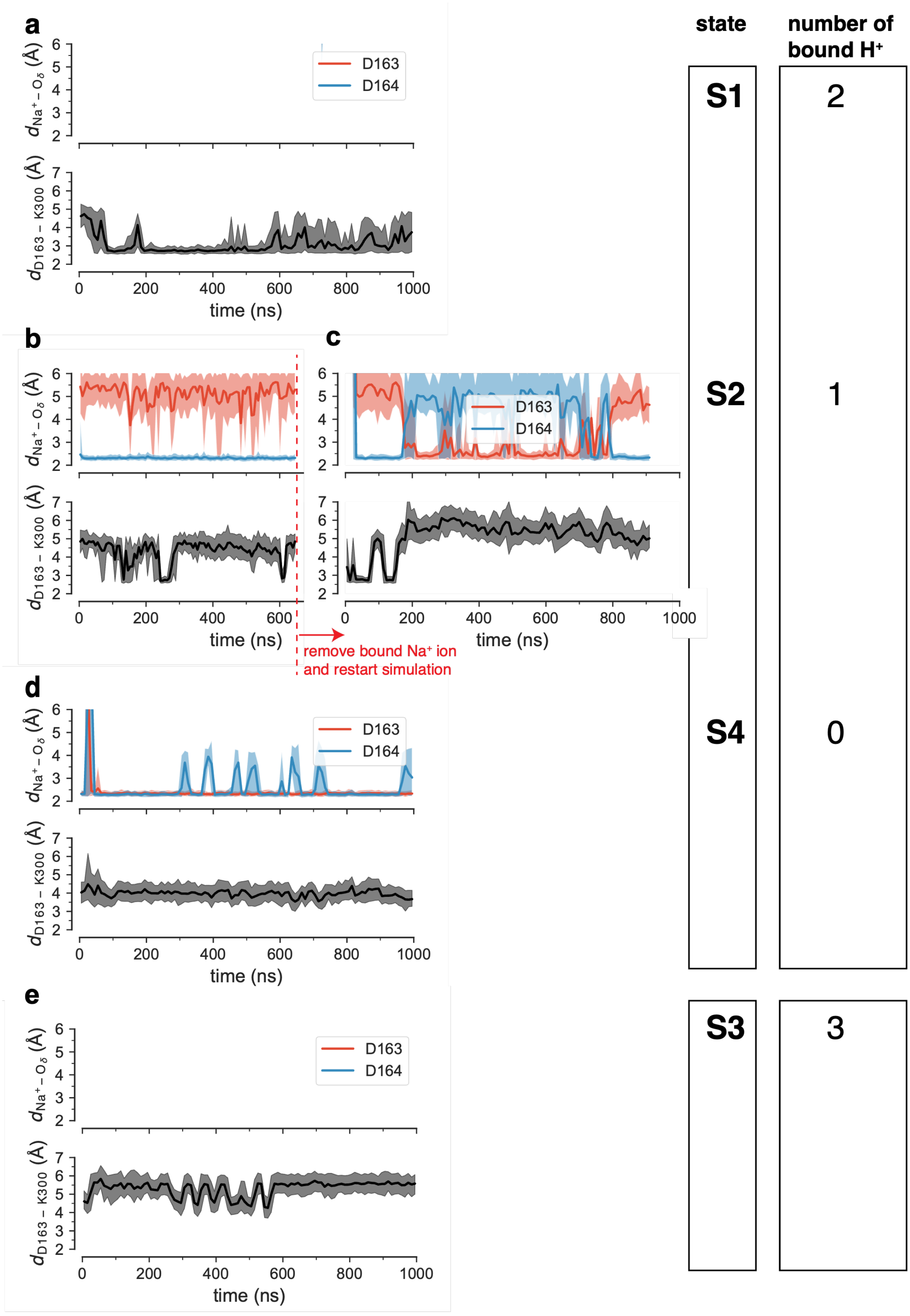
a
b
c
d
e
D163
D164
$d_{Na^+-O_\delta}$ (Å)
$d_{D163-K300}$ (Å)
time (ns)
remove bound Na⁺ ion
and restart simulation
state
number of bound H⁺
S1
2
S2
1
S4
0
S3
3

Supplementary Figure 8. Representative timeseries of binding site distances from MD simulations for differing protonation states. The top timeseries in each panel characterizes Na^+ binding and is the minimal Na^+ ion-carboxylate oxygen distance for the residues D163 (red) and D164 (blue). The bottom timeseries characterizes the D163-K300 salt bridge and is calculated as the minimal distance between a carboxylate oxygen of D163 and the amide nitrogen of K300. To make the noisy timeseries easier to visualize, data were averaged in 10 ns bins and the average for each bind was drawn as the solid line. The translucent band represents the data between the 5th and the 95th percentile. The columns on the right indicate the protonation state the putative number of protons bound. **a.** Protonation state S1 (D164 and K300 protonated, D163 deprotonated), run 0, protomer B. No ion binding was observed and hence no data are shown for the ion-aspartate distances. **b.** Protonation state S2 (D164 deprotonated, K300 protonated, D163 deprotonated), run 0, protomer A. A Na^+ ion was bound to D164 from the beginning and disrupted the D163-K300 salt-bridge. **c.** Same simulation as in (B) after manual removal of the bound Na^+ and restart of the simulation. The salt bridge re-formed in the absence of a bound Na^+. After <20 ns another Na^+ ion spontaneously binds and the salt-bridge breaks again. **d.** S4 state (D164, D163, and K300 deprotonated). **e.** S3 state (D164, D163, and K300 protonated), run 1, protomer A. The S3 configuration would only be realized at $pH < 2.5$ and is not considered relevant. No Na^+ binding is observed.

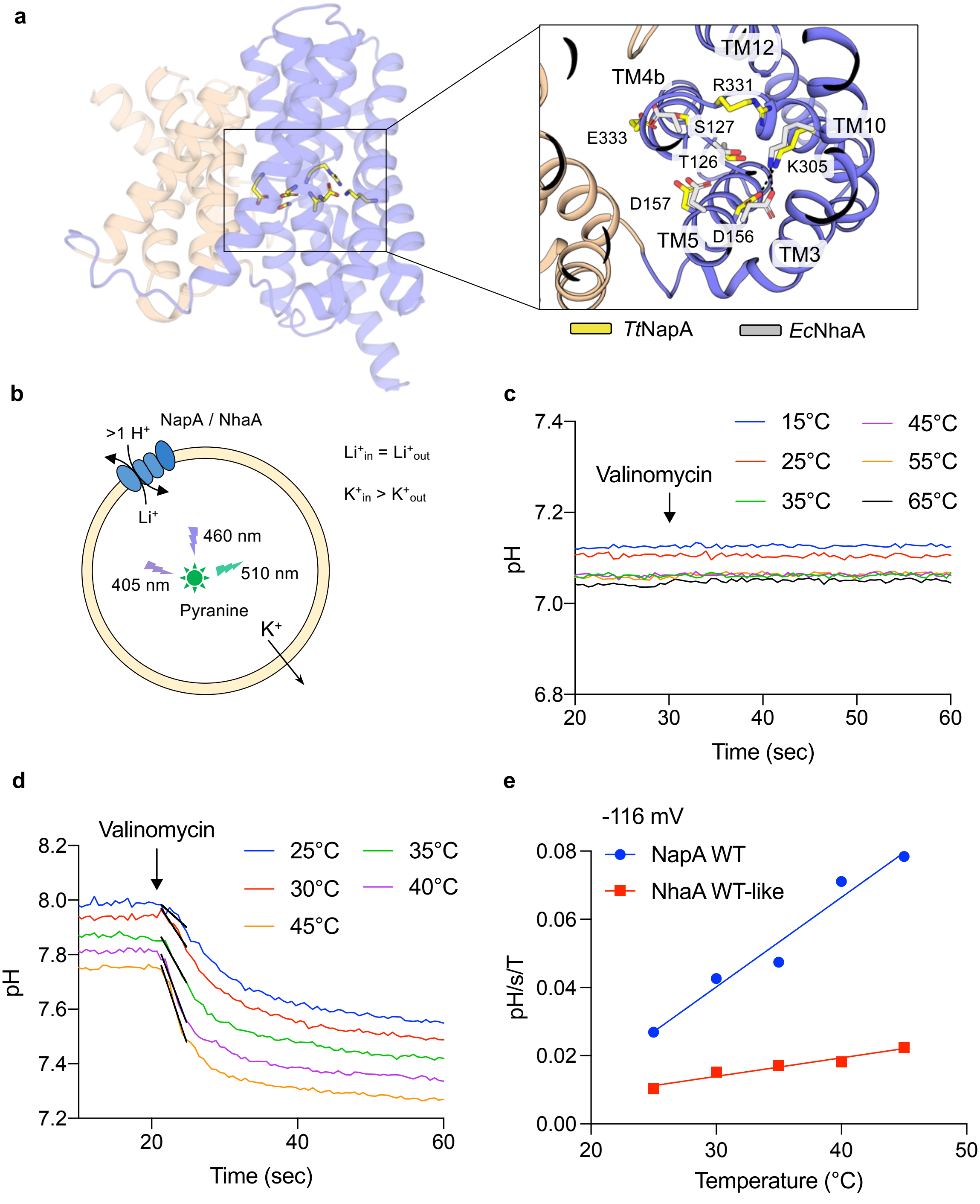

Supplementary Figure 9. Membrane potential driven transport and temperature-dependent rate determination of *Tt*NapA and *Ec*NhaA. a. Comparison of the ion-binding sites of NhaA and NapA, as cartoon with selected residues in sticks colored yellow and blue respectively and labeled. **b.** Schematic showing the experimental setup for membrane potential ($\Delta\Psi$) driven Li^+/H^+ exchange in proteoliposomes. At t = 0s, a membrane potential in the form of a K^+ gradient with otherwise equal buffer conditions in- and outside the proteoliposome is present. At t = 30s, addition of the K^+ selective ionophore valinomycin allows for efflux of K^+, and thus for electrogenic Li^+/H^+ exchange; the resulting pH change is detected with the

ratiometric dye pyranine. **c.** Example traces obtained with the setup from **b.** and inactive *Tt*NapA Asp157Asn measured at different temperatures, confirming temperature stability of proteoliposomes. **d.** Exemplary traces of ΔΨ-driven *Tt*NapA WT transport measured at different temperatures and linear regression through initial transport activity. **e.** Rates determined in **c.** and for *Ec*NhaA and *Tt*NapA WT plotted against temperature. A linear regression through these points is drawn to determine the temperature dependence of transport rates. Data presented are fluorescence ratio / min as an average of two technical repeats. Source data are provided as a Source Data file.

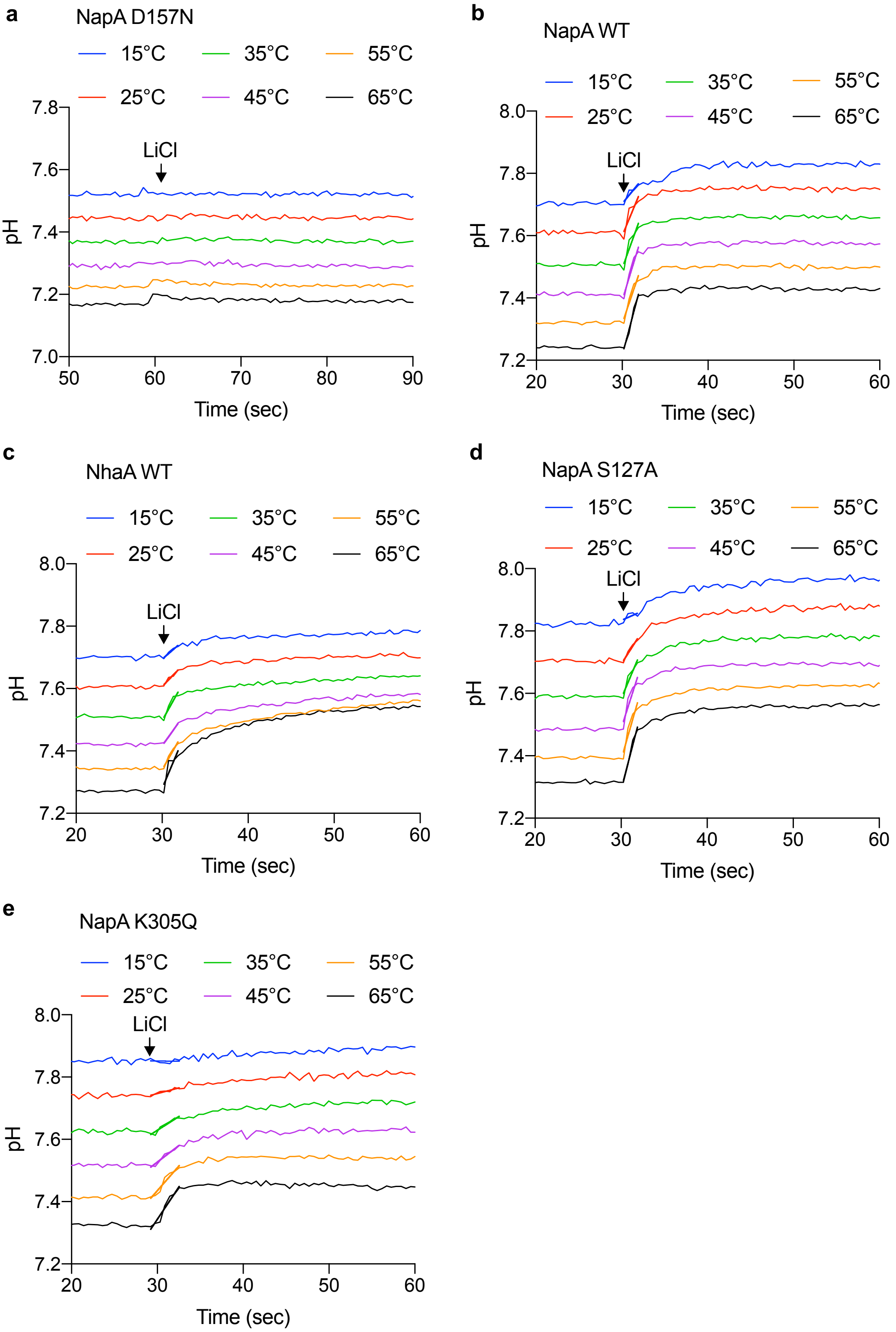

a
NapA D157N
15°C
35°C
55°C
25°C
45°C
65°C
LiCl
pH
7.8
7.6
7.4
7.2
7.0
50
60
70
80
90
Time (sec)
b
NapA WT
15°C
35°C
55°C
25°C
45°C
65°C
LiCl
pH
8.0
7.8
7.6
7.4
7.2
20
30
40
50
60
Time (sec)
c
NhaA WT
15°C
35°C
55°C
25°C
45°C
65°C
LiCl
pH
8.0
7.8
7.6
7.4
7.2
20
30
40
50
60
Time (sec)
d
NapA S127A
15°C
35°C
55°C
25°C
45°C
65°C
LiCl
pH
8.0
7.8
7.6
7.4
7.2
20
30
40
50
60
Time (sec)
e
NapA K305Q
15°C
35°C
55°C
25°C
45°C
65°C
LiCl
pH
8.0
7.8
7.6
7.4
7.2
20
30
40
50
60
Time (sec)

Supplementary Figure 10. Raw data traces obtained of substrate driven H^+ export. a. *Tt*NapA Asp157Asn dead mutant negative control showing that liposomes do not exhibit basal or substrate induced proton leak at higher temperatures at the relevant time-scale. **b-e.** Aliquots of the same variant are individually measured at different temperatures and the initial transport rate is determined by fitting a linear regression to the first 2-4 sec after substrate addition. Aliquots of the same sample are measured at different temperatures and the initial transport rate is determined by fitting a linear regression to the first 2-4 sec after substrate addition. Source data are provided as a Source Data file.

1 Huang, Y., Chen, W., Dotson, D. L., Beckstein, O. & Shen, J. Mechanism of pH-dependent activation of the sodium-proton antiporter NhaA. *Nat Commun* 7, 12940 (2016). https://doi.org:10.1038/ncomms12940

2 Winklemann, I. *et al.* Structure and elevator mechanism of the mammalian sodium/proton exchanger NHE9. *EMBO J* 39, e105908 (2020). https://doi.org:10.15252/embj.2020105908

3 Nji, E., Chatzikyriakidou, Y., Landreh, M. & Drew, D. An engineered thermal-shift screen reveals specific lipid preferences of eukaryotic and prokaryotic membrane proteins. *Nat Commun* 9, 4253 (2018). https://doi.org:10.1038/s41467-018-06702-3

Uncropped gel for Supplementary Figure 7b

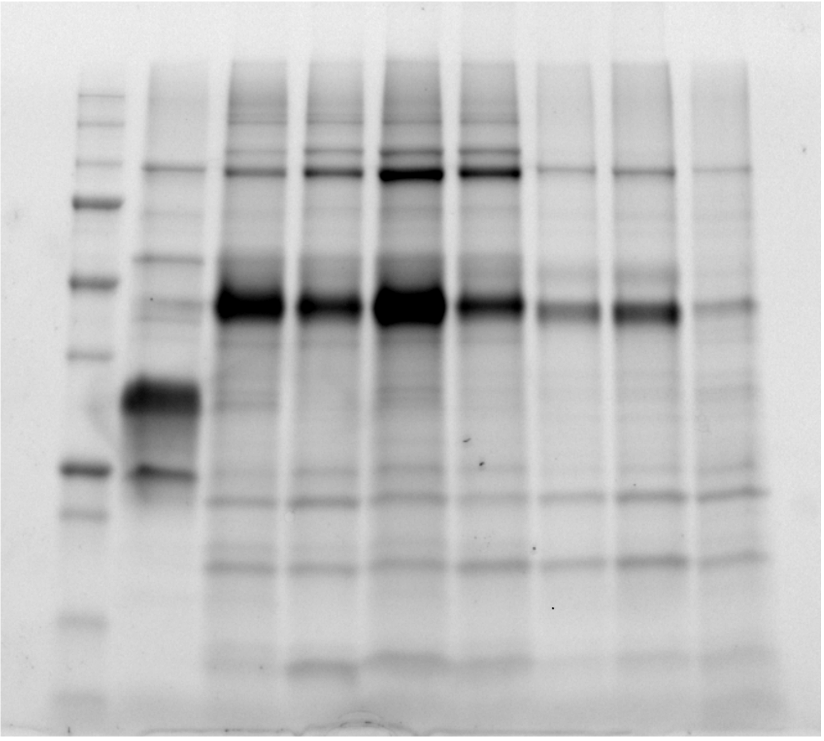

Supplement: Supplementary file 1 — Supplementary Information [file 41467_2022_34120_MOESM1_ESM.pdf]
