# Supplementary material for: Crystal structure of the Na+/H+ antiporter NhaA at active pH reveals the mechanistic basis for pH sensing
Source: Nat Commun. 2022 Oct 26;13:6383. doi: 10.1038/s41467-022-34120-z (PMC9606361; doi:10.1038/s41467-022-34120-z)
Supplement: Supplementary file 3 — Description of Additional Supplementary Files [file 41467_2022_34120_MOESM3_ESM.pdf]

File name: Supplementary Movie 1

Description: A morph between a cartoon representation of NhaA (dimer domain in light-blue; core domain in light-green) from the previous determined structure at inactive pH 3.8 (PDB ID: 4AU5, grey) to the newly determined structure at active pH 6.5 (PDB ID: 7S24, colored), as viewed from the cytoplasmic side. Key residues in the pH gating region (light-blue and light-green) and the strictly-conserved aspartic acid residue Asp164 (yellow) are also highlighted in stick-form.

File name: Supplementary Movie 2

Description: As in Supplementary Movie 1, but with an electrostatic surface representation also shown and rendered with 50% transparency.

File name: Supplementary Movie 3

Description: A morph between an electrostatic surface representation of NhaA structures at inactive pH 4.0 (PDB ID: 4AU5) and active pH 6.5 (PDB ID: 7S24). The ion-binding aspartate is indicated and shown as yellow sticks. Notably the ion-binding funnel open to the cytoplasmic side is much more open at active pH.
